# Magnetic Nanoparticle Arrays Self-Assembled on Perpendicular Magnetic Recording Media

**DOI:** 10.3390/ijms160819769

**Published:** 2015-08-20

**Authors:** Abdul Rahman Mohtasebzadeh, Longfei Ye, Thomas M. Crawford

**Affiliations:** 1 Smart State Center for Experimental Nanoscale Physics, Department of Physics and Astronomy, University of South Carolina, Columbia, SC 29208, USA; E-Mails: ramoh87@gmail.com (A.R.M.); lye@magassemble.com (L.Y.); 2 MagAssemble, Irmo, SC 29063, USA

**Keywords:** nanomanufacturing, self-assembly, magnetic nanoparticle, magnetophoresis, perpendicular magnetic recording, pattern transfer

## Abstract

We study magnetic-field directed self-assembly of magnetic nanoparticles onto templates recorded on perpendicular magnetic recording media, and quantify feature width and height as a function of assembly time. Feature widths are determined from Scanning Electron Microscope (SEM) images, while heights are obtained with Atomic Force Microscopy (AFM). For short assembly times, widths were ~150 nm, while heights were ~14 nm, a single nanoparticle on average with a 10:1 aspect ratio. For long assembly times, widths approach 550 nm, while the average height grows to 3 nanoparticles, ~35 nm; a 16:1 aspect ratio. We perform magnetometry on these self-assembled structures and observe the slope of the magnetic moment *vs.* field curve increases with time. This increase suggests magnetic nanoparticle interactions evolve from nanoparticle–nanoparticle interactions to cluster–cluster interactions as opposed to feature–feature interactions. We suggest the aspect ratio increase occurs because the magnetic field gradients are strongest near the transitions between recorded regions in perpendicular media. If these gradients can be optimized for assembly, strong potential exists for using perpendicular recording templates to assemble complex heterogeneous materials.

## 1. Introduction

Heterogeneous 3-dimensional nanocomposite materials have potential to transform human society, from advanced medicine to alternative energy. One way to create such materials is to assemble them from diverse nanoscale building blocks, *i.e.*, nanoparticles that serve as raw materials. Such nanoparticles could be directed to self-assemble into complex structures with heterogeneity on length scales from 100’s of nanometers to meters, spanning eight orders of magnitude in length. There are a plethora of self-assembly techniques that employ thermal or entropic forces to cause nanoparticles to assemble at larger length scales [1]. In addition, there are techniques that use externally-controlled forces, for example created by electric and magnetic fields, to direct self-assembly [2]. While Francis Bitter in essence used magnetic field-directed self-assembly to visualize magnetic domains in 1931 [3], since 2000 there has been a resurgence of interest in leveraging action-at-a-distance magnetic forces for assembly and control of micro- and nano-structures for applications that range from manufacturing to biomedicine [4,5,6,7,8,9]. Most of these efforts have focused on micrometer size ranges and use physical patterning of magnets to localize field gradients and direct assembly of micro- to nano-scale materials.

An alternative more akin to Bitter’s approach is to use the magnetically-patterned domains that are recorded to store information in a commercial disk drive as templates to assemble magnetic nanoparticles, *i.e.*, repurpose the disk drive for nanomanufacturing [10,11]. In contrast to using lithographic or other techniques to physically pattern magnetic materials, the disk drive already has the ability to magnetically-pattern large areas of recording media into distinct regions as small as 10 nm long and 30 nm wide, which is necessary to obtain storage densities of 1 trillion bits in a single square inch of recording media (Tbit/in^2^) [12,13]. While this type of self-assembly has been demonstrated with longitudinal recording [10], where the nanometer-sized Co grains that make up the medium have their magnetic moments oriented in the plane of the disk, it is difficult to realize fully-programmable 2D patterning with longitudinal media.

To keep increasing storage density the disk drive industry transitioned in 2005–2006 from longitudinal recording to perpendicular recording, where the Co magnetic moments are oriented perpendicular to the disk. This transition, critical to shrink bit sizes, maintain thermal stability, and still allow recording with an electromagnet, has the potential to allow nanoparticle self-assembly into user-programmed patterns with full-control in 2-dimensions, since the grain moments are perpendicular to the plane of the medium. While the Bitter technique has been used to characterize early perpendicular media at micrometer length scales [14], using perpendicular media to direct self-assembly at the nanoscale has yet to be explored.

In this paper, we study self-assembly of magnetic nanoparticles onto the surface of a perpendicular recording medium that is patterned into a series of parallel lines with 750 nm line spacing, *i.e.*, a nanomanufactured diffraction grating [15]. The assembled features and aspect ratios are characterized using electron and atomic force microscopy [16]. Further, using the Pattern Transfer Nanomanufacturing™ technique [11,15], we measure the magnetic properties of nanoparticle assemblies and correlate them with their physical properties [16]. The results suggest that by optimizing the magnetic properties of the nanoparticles and the recording medium, nanoscale control over feature size, shape, and aspect ratio may be possible. By using nanoparticles with a magnetic core and a metallic, semiconducting, or insulating shell [17,18], a variety of patterned, user-programmed composite materials could be assembled with nanometer precision using magnetically-recorded templates.

## 2. Results and Discussion

### 2.1. Feature Width

**Figure 1:**
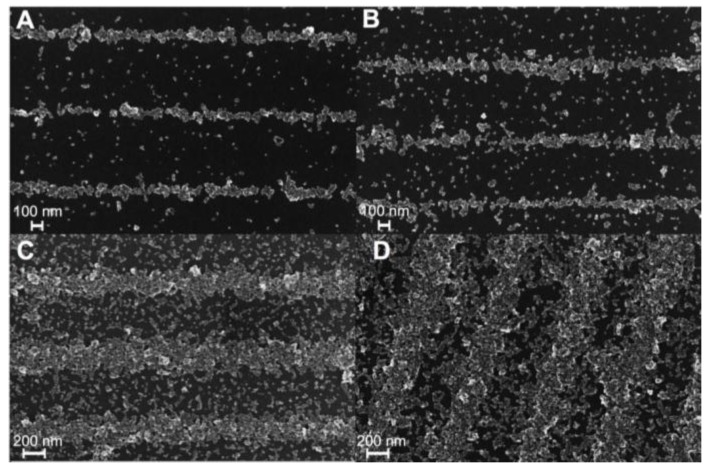
Scanning Electron Microscope (SEM) images of nanoparticle self-assembly onto perpendicular media recorded with 750 nm wide regions of alternating magnetization direction (up and down), for different coating times: (**A**) 5 min; (**B**) 15 min; (**C**) 30 min; and (**D**) 60 min. Note the significant change in width as a function of time as well as the assembly of material into the gaps with increasing coverage.

**Figure 2:**
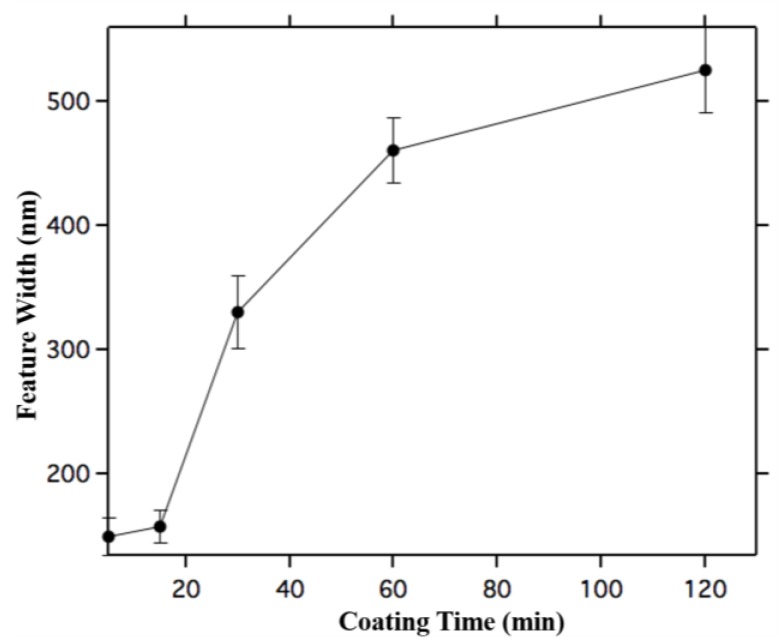
Average width of assembled nanoparticle features as a function of coating time. Error bars are 1 σ standard deviation taken by averaging many linescans obtained from the SEM image shown in Figure 1. Note that the width increases slowly at first, accelerates, and then slows down again.

Figure 1 A–D shows Scanning Electron Microscope (SEM) images of coupons coated for 5, 15, 30 and 60 min respectively. For both 5 and 15 min there are gaps between particles within the patterns, while these gaps are less noticeable for 30 and 60 min coatings, suggesting that features fill in and then grow in width as time progresses. There are also more non-assembled particles between features for 15 min than for 5 min. Figure 1C shows that while the widths at 5 and 15 min coatings are similar, the width nearly doubles for 30 min, *i.e.*, the growth accelerates between 5 and 30 min. This acceleration is shown in Figure 2, which shows average feature width *vs.* coating time. The average width is obtained from 15 different SEM images of features that are analyzed with imageJ software. The average width is similar for 5 and 15 min, ~150 nm wide (150 and 158 nm respectively). By 60 min the feature width increases to 460 nm and then to 525 nm after 120 min. Thus after a slow initial assembly process, the feature width grows rapidly from 15–60 min and slows again beyond.

### 2.2. Feature Height

**Figure 3:**
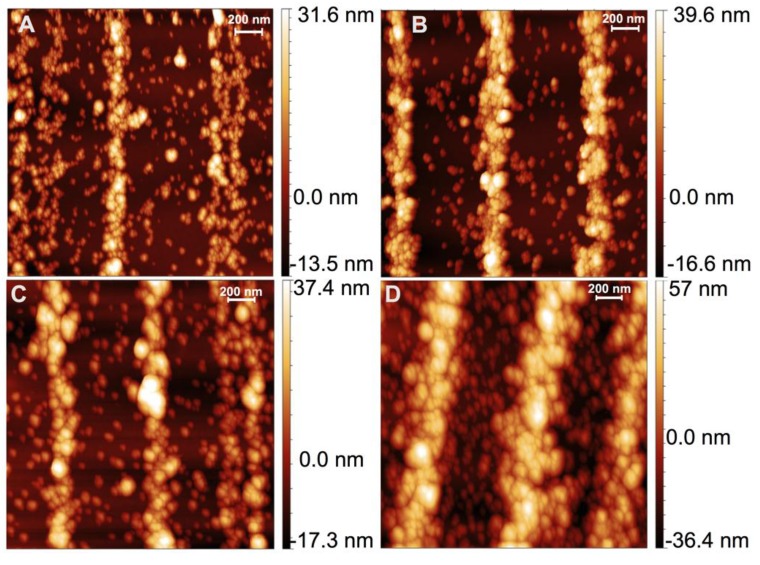
Atomic Force Microscopy (AFM) images of nanoparticle self-assembly onto the same features imaged with SEM in Figure 1, for different coating times: (**A**) 5 min; (**B**) 15 min; (**C**) 30 min; and (**D**) 60 min.

The SEM images in Figure 1 do suggest that the nanoparticle assemblies grow not only in width but also in height. To quantitatively measure feature height we employ Atomic Force Microscopy (AFM), as shown in Figure 3, where again A–D correspond to 5, 15, 30 and 60 min coatings times. For each coupon a cantilever with 300 kHz resonance frequency and 40 N/m force constant was used, and each coupon was scanned with 1024 pixel resolution in AC-Mode. Figure 3A shows an AFM image for a 5 min coating. In Figure 3 brighter features are higher. Similar to the SEM image in Figure 1A there are gaps within the patterns. Also, particles and crystals between arrays are noticeable in AFM imaging. We used Gwyddion software to subtract the background and analyze image line profiles from the AFM images.

**Figure 4:**
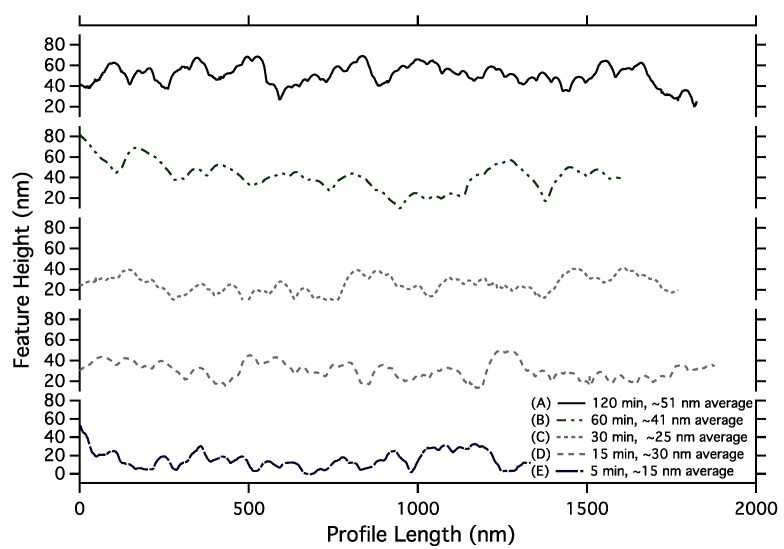
Vertical line profiles taken from AFM scans as in Figure 3: (A) 120 min; (B) 60 min; (C) 30 min; (D) 15 min; and (E) 5 min. The full scales of the feature height axis are 80 nm for all five profiles, showing the increase in height as coating time increases.

**Figure 5:**
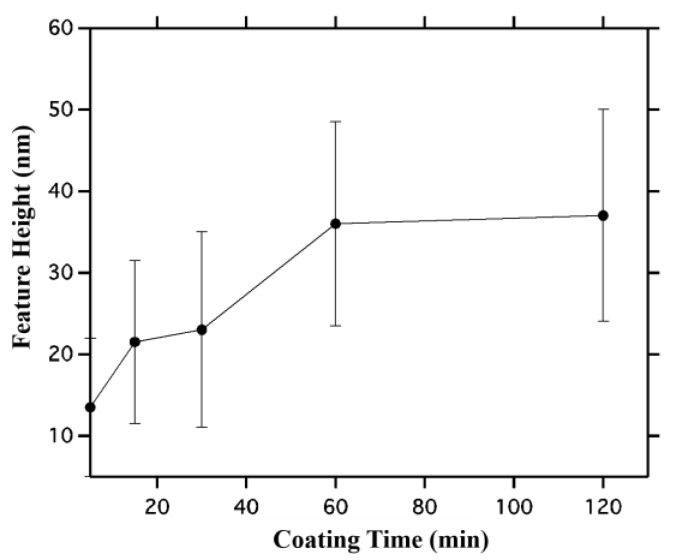
Average height of assembled nanoparticle features as a function of coating time. Error bars are 1 sigma standard deviation taken by averaging multiple linescans obtained from the AFM images shown in Figure 3. Interestingly, in comparison to the large increase in width observed in Figure 2, here the thickness appears to change from a single layer of nanoparticles, to two layers of nanoparticles, and finally to three layers of nanoparticles as time increases.

To obtain average height, vertical image line profiles (Figure 4 shows example vertical line profiles obtained from the AFM images for each coating time) were extracted from the AFM images from both assembled features and empty spaces between features. The average of empty space profiles was then subtracted from the pattern profiles to obtain feature height. Note that there are non-assembled nanoparticles in the empty space regions, and these are included in the average background because similar nanoparticles are likely present in all regions. Figure 5 shows average height as a function of coating time, with height changing from ~13 nm (about 1 nanoparticle) to ~22 nm (2 nanoparticles thick) at 15–30 min, and finally to 36 nm (3 nanoparticles thick). Note these are average values, and Figure 4 shows substantial variation in height along a feature, which is reflected in the error bars in the average height plotted in Figure 5.

The width and height data in Figure 2 and Figure 5 show that width increases faster than height. While the width grows from 150 nm at 5 min to 520 nm at 120 min, the height changes from 13 nm at 5 min to 37 nm at 120 min. Thus feature aspect ratio changes from 10:1 at 5 min to 14:1 at 120 min. After 5 min, nanoparticles appear to assemble on the transitions in random locations, yielding single nanoparticle heights on average, whereas for 120 min the average height is 3 nanoparticles. Since the standard deviation is larger for 120 min than 5 min, one source of increasing variation in feature thickness could be nanoparticle aggregation within the solution. After 5 min the assembling particles are not yet aggregated and thus single nanoparticles are assembled initially, while after 120 min nanoparticle aggregates are also being assembled.

### 2.3. Magnetic Properties of Patterned Arrays

**Figure 6:**
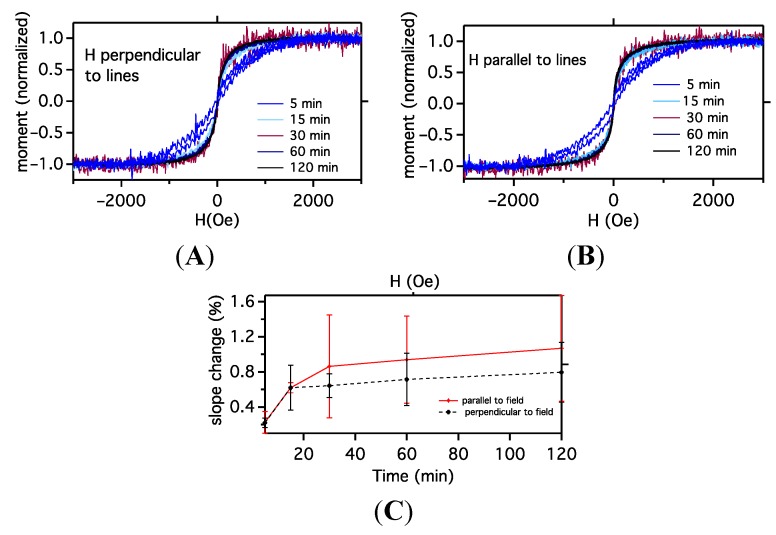
Magnetic moment as a function of external magnetic field for assembled nanoparticle patterns for different coating times measured: (**A**) with lines parallel to the applied field; and (**B**) with lines perpendicular to the magnetic field. Note the increase in slope as time increases. Since the slope change occurs for both orientations of magnetic field, it is likely not due to the increasing pattern width but due to clustering effects within the patterns, *i.e.*, not pattern–pattern interaction; Panel (**C**) shows the average slope of the m-H curve obtained at 20 and 100 Oe as a function of coating time, and while at long times, the perpendicular field loops have a lower slope than the parallel loops, the error bars overlap and thus beyond the slope change due to increasing feature size that is isotropic with direction, no difference between parallel and perpendicular fields can be claimed from the data.

Samples assembled for the same times as shown in Figure 1 and Figure 3 are pattern transferred to polymer films and mounted in two different orientations for magnetic characterization: grating lines perpendicular and parallel to the external field. Figure 6 A,B show normalized hysteresis loops for the five different coating times and different orientations. Interestingly, Figure 6A shows that the sample coated for 5 min has a smaller magnetic moment vs. magnetic field (mH) slope than the other four samples. As seen in Figure 6 A,B, the parallel and perpendicular m *vs.* H curves appear qualitatively similar. To determine whether there is a difference between parallel and perpendicular cases, the m *vs.* H curves were analyzed at low fields by fitting the linear part of the magnetization curves (the first term in a low-field expansion of the Langevin Equation is linear in magnetic field, *i.e.*, for low fields *L*~*a*/3, where *a* = µ_0_*mH*/K_b_*T*, and µ_0_ is the permeability of free space, *m* the magnetic moment, *H* the magnetic field, K_b_ is Boltzmann’s constant and *T* is the temperature) [16,19]. Thus we fit the linear region of the curves in Figure 6 A,B with *M*/*M*_0_ = *bH*, where M_0_ is saturation magnetization, M is magnetization at each field H and b is the slope, µ_0_*m*/3*K*_b_*T*. Average values of *b* (slope) and standard deviations for perpendicular and parallel cases are shown in Figure 6C. While the perpendicular and parallel slopes are identical at short times, the perpendicular has a smaller slope than the parallel for longer times. However, the standard deviations in Figure 6C suggest this difference is not statistically significant.

A changing m *vs.* H slope as a function of coating time is consistent with the SEM/AFM results which suggest that, at longer times, aggregates or small clusters dominate the assembly. Containing multiple interacting nanoparticle moments, these clusters are easier to magnetize than single particles, hence the larger slope (higher magnetic susceptibility). If magnetic interactions between aggregates dominate the magnetic behavior, then we would expect little difference between parallel and perpendicular orientations as observed.

## 3. Experimental Section

Commercial perpendicular recording media with standard magnetization and coercivity [20] was provided by HGST (Hitachi Global Storage Technologies) Inc. (San Jose, California, CA, USA) and was pre-recorded on a spin-stand [12]. The recorded pattern consists of 750 nm wide regions, which were alternately DC-erased with “up” and “down” magnetic fields using a commercial HGST perpendicular recording head. Thus the magnetization pattern is a 1500 nm period square wave along the radial direction of the disk, or a series of alternating moment cylindrical rings each 750 nm wide.

For these experiments we use diluted commercial Ferrotec (Nashua, NH, USA), EMG-707 ferrofluid. Dynamic Light Scattering measurements (Zetasizer, Malvern Inc., Worcestershire, UK) yield a nanoparticle hydrodynamic diameter ~90 nm, and ζ potential = −60 mV. TEM (H8000, Hitachi Inc., Tokyo, Japan) and Small Angle X-ray scattering measurements (Ultima IV, Rigaku Inc., Tokyo, Japan) yield a log-normal size distribution peaked at ~13 nm diameter, as typical of co-precipitation synthesized nanoparticles [21]. According to Ferrotec, EMG-707 has an effective bulk magnetization of 260 kA/m owing to a 0.83 nm thick dead layer on the nanoparticle surface [22]. 25 μL of EMG707 is added to 20 mL of 18 MOhm Deionized Water (DI) and vortexed for 10 seconds. Another 20 mL of DI water is added and mixed, followed by a final 10 mL of DI water and mixing, ultimately yielding 50 mL of dilute ferrofluid. EMG707 ferrofluid consists of ~0.001 percent volume concentration of ~12–13 nm diameter nanoparticles [11]. Finally <100 μL of Phosphate Buffered Saline (PBS) is added to the dilute ferrofluid [23]. We have observed that a specific amount of PBS enhances the self-assembly of nanoparticles observed through a dramatic increase in the intensity of light diffracted from all-nanoparticle diffraction gratings [15,24]. However, if larger amounts of PBS are added, the grating efficiency begins to decrease. This small volume of PBS does not cause significant aggregation of nanoparticles, as determined by Dynamic Light Scattering measurements [25], and allows the particles to remain colloidally suspended. However, it is important to start the nanoparticle assembly process at the same time (preferably earlier) after PBS is added, to maintain consistency from coating to coating, because eventually the nanoparticles will start to aggregate [25].

To assemble the nanoparticles, the surface of a 1 cm diameter “coupon” of magnetic media is cleaned with DI water and a cotton swab in a laminar flow clean hood to remove dust and other particles from its surface. This process is performed three times, with the final DI water rinse removed from the coupon’s surface by blowing with ultra-high-purity Nitrogen (Air Products, Allentown, PA, USA). The coupon is then sprayed with methanol and again wiped with a cotton swab. This process is repeated until the surface of the coupon is completely clean. We verify that the surface of the coupon is sufficiently clean by imaging it using a dark field optical microscope. To aid in verifying that the surface is clean, the coupon has a visible reference line (a scratch) which runs parallel to the recorded transition tracks on the surface of the media. The clean coupon is then mounted vertically on a holder attached to a precision rotation stage located above the beaker containing the dilute suspension. The coupon is lowered into the beaker and the rotation stage engaged to slowly translate and rotate the coupon through the suspension fluid for the desired coating time.

After the coating process is completed, the sample is immediately removed from the solution and dried. However, once the coupon is dried, some crystals inside the base suspension often remain on the surface of the coupon. To clean these crystals, a second beaker with pure DI water is located next to the coating beaker. However, one has to be careful not to wash the coated particles away. The coupon is dipped horizontally below the surface of the water but then removed vertically without shaking. Depending on the coating time, the coupon remains in the DI rinse for varying times from 1–6 s before removal. After rinsing, the coupon is allowed to remain vertical on a clean cotton cloth for 10–15 min. Once the coupon is coated and dried, the self-assembled magnetic nanoparticles form a diffraction grating that can be verified by tilting and observing the coupon in ambient light conditions.

Here we measure both the width and height of the self-assembled nanoparticle lines as a function of coating time, which is the time the colloidal suspension is allowed to remain on the coupon surface, with Scanning Electron Microscopy (Ultraplus FESEM, Zeiss Inc., Jena, Germany) and Atomic Force Microscopy (PicoPlus AFM, Agilent, Inc., Chandler, AZ, USA) respectively. For width analysis, 15 different images are obtained from multiple coatings and then average feature width obtained using imageJ. For height, linescans are taken along the top of a feature and along the coupon surface between features. These linescans are averaged and their difference taken to obtain the average feature height.

To determine how the assembly’s magnetic properties change with coating time, we employ a technology called Pattern Transfer Nanomanufacturing™, developed at the University of South Carolina, [11] to transfer the assembled nanoparticles from the media coupon surface to a standalone polymer film. First a liquid polymer solution is spin-coated onto the coupon’s surface. Diskcoat 4220 (General Chemical Corp., Brighton, MI, USA), is slightly diluted with DI water (one part DI water added to four parts Diskcoat) [23]. About 500 μL of liquid polymer solution is then spin-coated onto the coupon surface for 20 s at 2000 revolutions per minute (RPM). The polymer is cured under airflow in a hood for 25 min at room temperature. The thin polymer film will adhere to the self-assembled nanoparticles on the coupon surface and when the polymer is peeled from the coupon, it will transfer the nanoparticles from the coupon to the polymer [11]. To peel the polymer layer from the surface of the coupon, either double sided tape or adhesive binder reinforcement round tapes (which perfectly match the diameter of the coupon) are used.

The magnetic properties of polymer films (plus tape binder) with transferred patterns are measured in a Vibrating Sample Magnetometer (VSM) (Physical Property Measurement System, Quantum Design Inc., San Diego, California, CA, USA) with the external magnetic field aligned either parallel to or perpendicular to the grating lines. We measure each sample twice, first from −3000 to 3000 Oe with 11 Oe/s sweep rate, in order to see the change in magnetization hysteresis loop for each sample, and then we measure over a smaller 50 Oe range at 2 Oe/s to observe the slope of the *m vs. H* curve for smaller fields. All measurements are done at a constant temperature of 300 K.

## 4. Conclusions

We have employed SEM, AFM and VSM to study magnetic-field directed self-assembly of magnetic nanoparticles into patterned arrays on the surface of perpendicular magnetic recording media. Imaging shows that while both average width and height increase as a function of coating time, the aspect ratio of assembled features also increases, from 10:1 at 5 min to 14:1 at 120 min. Magnetometry shows an increase in susceptibility with increasing coating time meaning that it is harder to magnetize samples coated for 5 min than samples coated for 2 h. Compared with longitudinal magnetic recording [12] where the fields are emitted from the boundaries between recorded regions, in perpendicular recording, the stray fields originate from the entire recorded region and not the boundaries. Thus one might expect nanoparticle capture to occur at transitions between regions for longitudinal media [11] but over entire regions for perpendicular media. Our results clearly demonstrate that nanoparticles also assemble on the transitions for perpendicular media, as determined by the spatial dependence of the magnetic field gradients above perpendicularly magnetized bits. As seen from Magnetic Force Microscopy (MFM) images of perpendicular media using a novel technique capable of separating in-plane and out-of-plane field gradients [26], as well as by computing the gradients of the standard solution to Laplace’s equation for the stray field emitted by a periodic array of perpendicular bits [27,28].

(1) $$H_{\text{z}}=\frac{32M_{\text{s}}}{\text{π}}\sum_{n,odd}\underset{k,odd}{\sum }\frac{1}{nk}\text{sin}\left(\frac{n\text{π}}{a}x\right)\text{sin}\left(\frac{k\text{π}}{b}y\right)\times \left\{1-exp\left[-\text{π}{\left(\frac{n^{2}}{a^{2}}+\frac{k^{2}}{b^{2}}\right)}^{\frac{1}{2}}\text{δ}\right]\right\}\times exp\left[-\text{π}{\left(\frac{n^{2}}{a^{2}}+\frac{k^{2}}{b^{2}}\right)}^{\frac{1}{2}}\left|z\right|\right]$$

where *M*_s_ is the medium saturation magnetization, and *a* and *b* are the dimensions of a bit along *x* and *y* respectively, *z* is the height above the medium, and δ is the medium thickness. The *z*- and *x*- components of the gradient of *H*_z_ are largest at (*x*-gradient) or near (*z*-gradient) the transitions between opposing magnetic regions and they are much smaller in the middle of a bit. Thus as the nanoparticles approach the surface, they are attracted to the larger gradients at the transitions. The aspect ratios we observe as a function of coating time suggest that the *x*-directed gradient [27,28] causes lateral feature growth to dominate over vertical growth. Given the presence of a destabilizing salt, nanoparticle aggregation in the bulk fluid and in the field gradients above a transition could lead to larger magnetic moments and hence larger forces on aggregates, modifying the assembly properties. While one might hypothesize that at the longest coating times the macroscopic line features would begin to magnetically interact with each other, the similarity of magnetization curves between parallel and perpendicular field alignment suggests instead that the dominant interaction is local within a pattern, perhaps transitioning from particle–particle to aggregate–aggregate interaction. One way to separate interactions between arrays is to measure magnetic properties at lower temperatures, where a larger magnetic moment would enhance the interactions between lines [16]. Additional imaging and magnetometry studies, especially as a function of PBS volume added before assembly and as a function of temperature, are required to better elucidate the nature of local nanoparticle–cluster magnetic interactions in these nanocomposite assemblies.
